# Malaria and vitamin A deficiency in African children: a vicious circle?

**DOI:** 10.1186/1475-2875-8-134

**Published:** 2009-06-17

**Authors:** Miguel A SanJoaquin, Malcolm E Molyneux

**Affiliations:** 1 Malawi-Liverpool-Wellcome Trust Clinical Research Programme, College of Medicine, Po Box 30096, Chichiri, Blantyre 3, Malawi; 2 Liverpool School of Tropical Medicine, Liverpool, UK

## Abstract

Vitamin A deficiency and malaria are both highly prevalent health problems in Africa. Vitamin A deficiency affects over 30 million children, most of whom are in the age-group (under five years) most affected by malaria. Vitamin A deficiency increases all-cause mortality in this part of the population, and malaria is an important cause of death in children at this age. A low serum retinol concentration (a marker of vitamin A deficiency) is commonly found in children suffering from malaria, but it is not certain whether this represents pre-existing vitamin A deficiency, a contribution of malaria to vitamin A deficiency, or merely an acute effect of malaria on retinol metabolism or binding. In this paper, available evidence in support of a causal relationship in each direction between vitamin A deficiency and malaria is reviewed. If such a relationship exists, and especially if this is bidirectional, interventions against either disease may convey an amplified benefit for health.

## Background

Malaria commonly afflicts populations that are both impoverished and malnourished, and a large proportion of the burden of malaria falls upon children. There were more than 100 million malaria episodes among young children in sub-Saharan Africa in the year 2000, according to a recent estimate [1]. Malaria is a preventable and easily treatable disease, yet ~800,000 children die from it in Africa every year [2]. Malaria is the commonest reason for hospitalization among children and it is a leading contributor to the widespread problem of anaemia [3].

Vitamin A is an essential nutrient required for maintaining immune function, playing an important role in the regulation of cell-mediated immunity and in humoral antibody responses [4-6]. Vitamin A deficiency (VAD) is an extended public health problem, especially in Africa [7]. Preschool-age children and women of reproductive age are the two population groups most at risk. Vitamin A supplementation has been shown to decrease the incidence of measles, diarrhoeal disease and all-cause mortality, as well as to improve several aspects of eye health [8-14].

The relationship between malaria and VAD deserves careful scrutiny. The available evidence associating VAD with malaria is reviewed and discuss whether this association may be causal and bidirectional.

### Vitamin A sources and storage

Two main dietary forms of vitamin A exist: pre-formed vitamin A, found in foods such as butter, egg yolks and cod-liver oil, and provitamin A carotenoids, found in foods such as spinach, carrots, mangoes and papayas. Approximately 90% of the vitamin A in the body is stored in the liver, and an adult liver can contain enough vitamin A to last more than 1 year. In developing countries a lower than required dietary intake of vitamin A-rich foods commonly results in deficiency, especially among young children [15].

### Measuring vitamin A status

The most accurate measure of an individual's vitamin A status is the hepatic vitamin A content. To assess this requires an invasive technique that is impractical for population studies. Alternative methods for estimating VAD at population level include measuring breast milk vitamin A, dark adaptometry, pupillary responses, conjunctival impression cytology and serum or plasma retinol concentration [16]. Of these the latter has been most widely used, especially in population studies, as an accessible indicator of vitamin A status [17]. A circulating retinol concentration of <0.70 μmol/L (<20 μg/dl) is usually taken to indicate deficiency in children [16]. The limitations of this method, especially in the context of acute infection [18], are discussed below.

### Community prevalence of VAD

**Figure 1:**
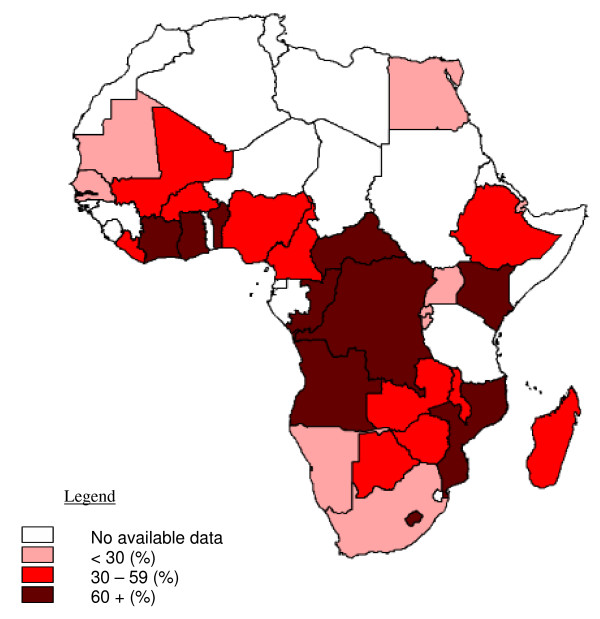
**Distribution of vitamin deficiency in African pre-school children**. Vitamin A deficiency is defined as serum retinol <0.70 μmol/L. These data are broadly based on surveys conducted during the year 2000 and 2003.

The prevalence of VAD (defined as serum retinol <0.70 μmol/L or the presence of abnormal impression cytology) among African pre-school children in the year 2000 was estimated to be 32%, affecting 33 million children (Micronutrient Deficiency Information System [MDIS] of the World Health Organization)[19]. The same and linked studies revealed a prevalence of xerophthalmia of 1.5%, affecting an estimated 1.5 million children [17]. There is a scarcity of more recent information by which to assess whether the prevalence of VAD has fallen in subsequent years during which there have been large-scale vitamin A supplementation campaigns. Figure 1 shows the most updated information on VAD for Africa using the database generated by WHO with some more recent national surveys [19]. Where an age-breakdown is provided, as in Malawi, VAD prevalence is highest among young children (~60% in children aged 6–36 months) and it decreases in older ages (~40% in children aged 6–12 years).

### Possible mechanisms of enhanced malaria susceptibility in VAD

The immune response against malaria involves both innate and specific acquired immune mechanisms [20]. It has been suggested that VAD impairs antibody response to malarial antigens that require Th2-mediated help [21]. Vitamin A acts as a regulator of more than 300 genes through its active metabolite, all-trans and 9-cis retinoic acid, and specific nuclear receptors that are in the steroid and thyroid hormone receptor superfamily [22]. Serghides et al [23] investigated the effects of 9-cis-retinoic acid, a metabolite of vitamin A, on CD36 expression, non-opsonic phagocytic clearance of parasitized erythrocytes, and TNFα production in human monocytes and macrophages. The metabolite reduced secretion of TNFα, upregulated CD36 expression, and increased phagocytosis of *Plasmodium falciparum*-parasitized erythrocytes. The authors concluded that increased parasite clearance and reduced proinflammatory cytokine responses to infection might partly explain the beneficial effects of supplementation with vitamin A in malaria.

### Does vitamin A supplementation decrease the risk of malaria?

Approximately two thirds [24] of the 10.8 million child deaths [25] that currently occur can be prevented by available interventions, of which vitamin A supplementation (VAS) is one. A meta-analysis of several large vitamin A trials has shown that improving vitamin A status reduces overall mortality rates by 23–34% among children six months to five years of age if vitamin A supplementation is given at least twice per year at coverage rates of at least eighty percent [8,26,27]. The reduction in mortality was particularly notable for measles (33–78%) and diarrhoea (24–50%). The effect of vitamin A (% reduction in mortality) does not seem to be influenced by age or gender in children aged six months to five years. The most logical explanation for these observations is that VAD increases overall mortality and that it predisposes to some cause-specific deaths.

Is malaria one of the conditions aggravated by VAD? Binka *et al *[28] first investigated this question in two companion, randomized, placebo controlled trials of the impact of vitamin A supplementation on malaria parasitaemia, morbidity, and mortality in young children (aged 6–59 months) in northern Ghana. In the mortality study 21,906 children were visited every four months over a period of two years, and in the morbidity study 1,455 children were visited weekly for one year. The vitamin A dosing regime was the same in the two trials: children aged 6–11 months were randomized to receive either 30 mg retinol or placebo and older children to receive either 60 mg retinol or placebo every four months. Analysis of outcomes showed a 20% reduction in all-cause mortality in the groups receiving vitamin A, but no difference in identifiable malaria-specific mortality rates (RR = 1.03; 95% CI 0.74, 1.43). Similarly, there was no evidence in this study that supplementation reduced the incidence of probable malarial fever (reported symptoms with no slide confirmation), malaria parasitaemia or mean parasite density in children with a positive blood smear. The investigators reported that there was no correlation between serum retinol concentration at the beginning of the trial and subsequent malaria parasite density in children who received placebo (r = 0.01). It was then concluded that vitamin A supplementation had no impact on malaria in this population, despite the 20% reduction in overall mortality.

Different findings were reported in two subsequent randomized controlled trials assessing the impact of vitamin A supplementation on slide confirmed malaria. In a study conducted in Papua New Guinea [29] 480 children aged 6–60 months were randomly assigned to receive high-dose vitamin A or placebo every 3 months for 13 months. Malaria morbidity was assessed both through weekly community-based case detection and by surveillance of patients who self-reported to the health centre. The number of episodes of fever with parasitaemia (temperature 37.5°C with a parasite count of at least 8000/μl) was 30% lower in the vitamin A group than in the placebo group (RR= 0.70; 95% CI 0.57, 0.87). Supplementation also reduced the mean parasite density in these symptomatic cases, but had no effect on the incidence of *P falciparum *infection (parasitaemia of any density, irrespective of symptoms) or anaemia. A recent randomized controlled trial conducted in Burkina Faso [30] assessed the impact of a single dose of 200,000 IU of vitamin A with daily zinc supplementation for a period of six months on 148 children aged 6–72 months. Children in the supplemented group had a significant 30% reduction in slide-confirmed malaria fevers.

### Does malaria contribute to VAD?

Two major methodological challenges complicate any attempt to assign to malaria a causal role in the development of VAD. These are: (i) the lack of enough evidence from prospective studies demonstrating that infection precedes deficiency; and (ii) the fact that the serum retinol concentration (the widely-used marker for vitamin A status) may be affected as part of the host's acute phase response to infection.

Associations have been reported between xerophthalmia (the severest clinical manifestation of VAD) and a variety of preceding acute illnesses, including diarrhoea, pneumonia and measles [31]. In one prospective study, children who had suffered from diarrhoea or respiratory disease were more likely to develop xerophthalmia subsequently [32]. These findings suggest that a reduction in the serum retinol concentration may follow a variety of acute infections, presumably mediated by a common host response.

More data are available for this effect in malaria than in other infections. In a recent large case-control study [33] in Malawi, 92% of 247 severe anaemia cases (mostly severe malarial anaemia) had low levels of serum retinol versus 66% of 262 controls. This study also detected an inverse association between serum retinol concentration and malaria parasite density (unpublished data) which is consistent with previous observations [34]. Other studies also found that serum retinol levels were substantially lower in individuals with malaria illness than in their healthy peers. In a case control study [35] involving 454 pre-school age Congolese children, 37.5% of the children suffering from malaria had plasma levels of retinol lower than 10 μg/dl (0.3 μmol/L). The mean concentration of plasma retinol in patients during malarial attacks (14.8 +/- 9.5 μg/dl; or 0.5 +/- 0.3 μmol/L) was significantly lower than the values found in control subjects (31.5 +/- 14.3 μg/dl; or 1.1 +/- 0.5 μmol/L) (p < 0.001). In seven non-immune French adults suffering from malaria [36], serum retinol levels were lower than in controls and correlated inversely with the density of parasitaemia (r = -0 338, P = 0.035). In 24 Vietnamese adults with severe malaria, serum retinol concentrations during the acute illness were lower than in healthy controls, and increased significantly during the first week after the start of treatment [37]

Investigations of this kind cannot demonstrate causality nor its direction, and none of these studies evaluated vitamin A status previous to the infection. An indirect attempt to determine antecedent VAD in patients with malaria was made in a study in Thailand, in which the mean serum retinol concentration was lower in children with malaria than in controls [38]. In this study the investigators did not find evidence of clinical vitamin A deficiency in the communities studied; they speculated, without convincing evidence, that severely depleted stores of retinol were unlikely in the patients presenting with malaria. The authors argued that the low serum retinol levels seen might be attributed to the acute phase response of the infection. This possibility is discussed below.

### Mechanisms by which malaria could contribute to VAD

The observation that clinical malaria or even asymptomatic parasitaemia is associated with low serum retinol levels seems consistent across the studies referred to above. However, it remains controversial whether the reduction in retinol levels reflects an actual depletion of vitamin A in the liver. A number of possible mechanisms have been proposed by which malaria could lead to VAD [5].

#### Decreased intake

Sick children tend to eat less, and malaria could precipitate VAD in a child who is already malnourished and has precarious hepatic reserves of vitamin A. This effect may be enhanced by the fact that the malaria season is commonly a time when food supplies are scarce. There is evidence that serum carotenes are depressed in malaria patients independently of serum retinol concentrations, suggesting preceding reduced oral intake of carotene-containing compounds [38].

#### Malabsorption

There is an association between helminth infections and malaria [39] and it has been suggested that helminth infections may impair vitamin A uptake [40].

#### Direct loss

Urinary retinol losses during severe infection can be substantial. A study showed that adults in intensive care with pneumonia and sepsis lose nearly three times the recommended dietary allowance, whereas healthy adults lose <1% of the recommended dietary allowance per day [41]. Impaired tubular reabsorption of low-molecular weight proteins, including retinol-binding protein (RBP) may be responsible for this [41,42]. It is not known whether or to what extent malaria has this effect.

#### Impaired plasma carrying capacity

As part of the acute phase response [43] cytokines trigger the induction of fever and production of cortisol and, in the liver, increase the production of positive acute-phase proteins while decreasing the production of negative acute-phase proteins, including RBP [44]. RBP is the binding protein by which vitamin A is transported from the liver to the tissues where it is required.

Is the plasma retinol concentration during an acute infectious illness of any value as an indicator of an individual's vitamin A status? A prospective study [45] assessing the clinical and laboratory measurements at admission and recovery of 90 children with dysentery (66 with shigellosis) hospitalized in Bangladesh showed that serum retinol concentrations were low at admission, but were significantly greater at discharge even though no vitamin A supplements were given during the illness (0.36 +/- 0.22 compared with 1.15 +/- 0.50 μmol/L; or 10.3 +/- 6.3 compared with 33 +/-14.3 μg/dl P < 0.001). The extent of this increase was significantly associated with severity of disease and was greatest in children with poor underlying nutritional status, particularly those with a low weight-for-age. In Indian children with measles, it took eight weeks on average after recovery from the acute illness for normal concentrations of retinol and retinol binding protein to be restored [46]. There seems to be no data showing whether or how soon retinol levels are restored after a malaria infection has been eliminated, or of any investigations of the possibility that the low retinol levels in these circumstances, even if transient, may further increase susceptibility to malaria or other infections.

## Conclusion

The available evidence suggests that vitamin A supplementation is a good strategy for children living in malaria-endemic regions, because deficiency is common and because supplementation reduces all-cause mortality. Supplementation also reduces the incidence of vitamin A-specific pathology such as xerophthalmia. Although vitamin A supplementation reduces the incidence of uncomplicated malaria by about one-third, it does not appear to reduce the rate of deaths that can be specifically attributed to malaria. Evidence is weak for malaria having a causal role in the development of VAD, although on theoretical grounds and from indirect evidence this is a distinct possibility.

Current programmes that deliver separate interventions against malaria and VAD may have a synergistic effect, and coordination of these interventions may provide added benefit against both diseases.

## Abbreviations

RBP: Retinol binding protein; RR: Relative risk; VAD: Vitamin A deficiency; VAS: Vitamin A supplementation; WHO: World Health Organization.

## Competing interests

The authors declare that they have no competing interests.

## Authors' contributions

MAS performed the literature search, reviewed all articles cited and drafted the manuscript. MEM confirmed the findings and conclusions, and provided additional commentary and perspective. All authors contributed to and approved the final manuscript.
